# Preparation of Monolithic LaFeO_3_ and Catalytic Oxidation of Toluene

**DOI:** 10.3390/ma16113948

**Published:** 2023-05-25

**Authors:** Songlin Han, Yaqiu Tao, Yunfei Liu, Yinong Lu, Zhigang Pan

**Affiliations:** 1 College of Materials Science and Engineering, Nanjing Tech University, Nanjing 211800, China; 202061203219@njtech.edu.cn (S.H.); taoyaqiu@njtech.edu.cn (Y.T.); yfliu@njtech.edu.cn (Y.L.); yinonglu@njtech.edu.cn (Y.L.); 2 State Key Laboratory of Materials-Oriented Chemical Engineering, Nanjing 211800, China

**Keywords:** LaFeO_3_, monolithic, porous material, toluene, catalytic oxidation

## Abstract

Porous LaFeO_3_ powders were produced by high-temperature calcination of LaFeO_3_ precursors obtained by hydrothermal treatment of corresponding nitrates in the presence of citric acid. Four LaFeO_3_ powders calcinated at different temperatures were mixed with appropriate amounts of kaolinite, carboxymethyl cellulose, glycerol and active carbon for the preparation of monolithic LaFeO_3_ by extrusion. Porous LaFeO_3_ powders were characterized using powder X-ray diffraction, scanning electron microscopy, nitrogen absorption/desorption and X-ray photoelectron spectroscopy. Among the four monolithic LaFeO_3_ catalysts, the catalyst calcinated at 700 °C showed the best catalytic activity for the catalytic oxidation of toluene at 36,000 mL/(g∙h), and the corresponding T_10%_, T_50%_ and T_90%_ was 76 °C, 253 °C and 420 °C, respectively. The catalytic performance is attributed to the larger specific surface area (23.41 m^2^/g), higher surface adsorption of oxygen concentration and larger Fe^2+^/Fe^3+^ ratio associated with LaFeO_3_ calcined at 700 °C.

## 1. Introduction

Volatile organic compounds, abbreviated as VOCs, are compounds with a low boiling point at ambient temperature and pressure [1]. VOCs are a large class of carbon-based compounds, most of which are diffusible, flammable, explosive or toxic. VOCs are considered to be the main sources of atmospheric pollution, posing serious ecological and human health hazards [2,3,4,5]. Such pollutants are produced either naturally or by human activities. Pollution due to human activities can be divided into two types: indoor and outdoor. Outdoor sources account for a large proportion of pollution from chemical plants, power plants, pharmaceutical plants, refineries and gas stations, as well as food processing, automobile manufacturing, furniture and textiles [6,7,8]. Volatile organic compounds are also harmful pollutants indoor air [9]. The main sources of indoor VOCs are solvents, glues, insulation materials, cooking and smoking waste [10,11,12]. When VOCs are emitted into the atmosphere, these high concentrations of VOCs can cause nausea, dizziness and headaches, some damage to human organs as well as the nervous system and even chronic or acute poisoning, leading to cancer in severe cases [13,14]. Decomposition of VOCs into non-toxic compounds is important for our environment.

Typical methods to deal with VOCs are condensation [15], adsorption [16], absorption [17], plasma technology [18], catalytic oxidation [19,20,21,22] and biodegradation [23]. Among these methods, catalytic oxidation technology converts volatile organic compounds directly into water and carbon dioxide without causing secondary pollution to the environment. The catalysts that have been used for environmental purposes are noble metal catalysts and metal oxide catalysts. Although noble metal catalysts show high catalytic activity for VOCs, their use is limited due to the high price and low availability [24]. Based on this situation, metal oxide catalysts are regarded as a good alternative to the noble metal catalyst due to their low price, ease of preparation, stability and good resistance to toxicity [25]. Perovskite oxides have been extensively studied in the catalytic oxidation of VOCs and have shown high catalytic activity during the oxidation process [26,27]. LaFeO_3_ is a typical perovskite that is widely used for catalytic oxidation [28,29,30,31] and gas and biosensors [32,33,34] due to its good catalytic activity and high stability. It is also used in solid oxide fuel cells [35] by virtue of its excellent catalytic activity, high electrical conductivity and oxygen mobility. To achieve a high catalytic performance, the LaFeO_3_ catalyst with a porous structure is desirable since porous materials possess a higher specific surface area. At the same time, monolithic catalysts have advantages over powder catalysts, such as better thermal conductivity and lower reactor pressure [36]. Therefore, the rational design of a monolithic catalyst with porous structure, high stability and good catalytic activity is of great significance.

**Figure 1:**
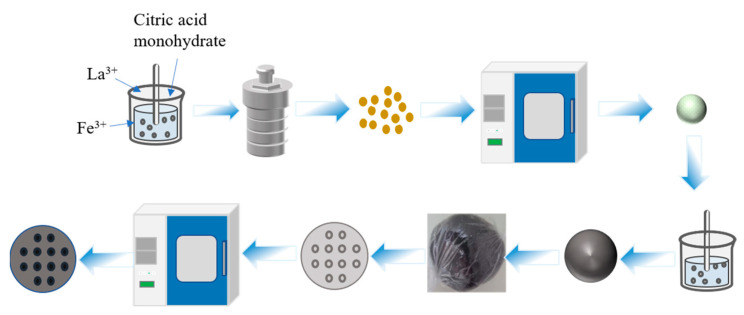
Preparation process of monolithic LaFeO_3_ catalysts.

In this study, powder LaFeO_3_ was synthesized by conventional high temperature calcination of LaFeO_3_ precursors, which were obtained using a simple hydrothermal route. Monolithic LaFeO_3_ catalysts were then prepared by mixing with kaolinite, carboxymethyl cellulose, glycerol and active carbon followed by a single extrusion method. Four monolithic LaFeO_3_ catalysts were prepared using LaFeO_3_ powders calcinated at 500 °C, 600 °C, 700 °C and 800 °C. The flow chart of powder LaFeO_3_ preparation is shown in Figure 1. The structure and physical and chemical properties of each catalyst were investigated using techniques including powder X-ray diffraction (XRD), scanning electron microscopy (SEM), X-ray photoelectron spectroscopy (XPS) and Brunauer–Emmett–Teller (BET) analysis. The monolithic 700 °C LaFeO_3_ catalyst with a large specific surface area, a high Fe^2+^/Fe^3+^ ratio and an abundant concentration of adsorbed oxygen exhibits excellent catalytic activity.

## 2. Materials and Methods

### 2.1. Materials

Ferric nitrate hydrate (Fe(NO_3_)_3_·9H_2_O) was purchased from Sinopharm Chemical Reagent Co. Lanthanum Nitrate Hexahydrate (La(NO_3_)_3_·6H_2_O) was supplied by Shanghai Aladdin Biochemical Technology Co. Citric acid monohydrate (C_6_H_8_O_7_·H_2_O) was purchased from Shanghai Lingfeng Chemical Reagent Co. Sodium Carboxymethyl cellulose ([C_6_H_7_O_2_(OH)_2_OCH_2_COONa]n) was provided by Sinopharm Chemical Reagent Co. Glycerol (C_3_H_8_O_3_) was provided by Shanghai Lingfeng Chemical Reagent Co. Kaolin (Al_2_O_3_·2SiO_2_·2H_2_O) was bought from Henan Plutonium Casting Material Co. Carbon (C) powder was provided by Tianjin Lihua Jin Chemical Co. All chemical reagents are of analytical grade and used without further purification.

### 2.2. Synthesis of LaFeO_3_ Powders

The powder LaFeO_3_ perovskite catalyst was synthesized by a hydrothermal method. Next, 1.299 g La(NO_3_)_3_·6H_2_O, 2.424 g Fe(NO_3_)_3_·9H_2_O, and 3.78 g citric acid monohydrate were added to 60 mL of deionized water with a molar ratio of n(La):n(Fe):n(citric acid) = 1:1.5:6 molar ratio and stirred for 30 min to yield a clear solution. The solution was transferred to a 100 mL PTFE-lined stainless-steel autoclave and kept at 180 °C in an oven for 8 h. The resulting solid yellow product was filtered and washed several times with distilled water and anhydrous ethanol. The resulting yellow product was dried at 80 °C for several hours and ground followed by calcination at appropriate temperatures. The final product calcinated at 500 °C, 600 °C, 700 °C and 800 °C, is denoted as 500 LFO, 600 LFO, 700 LFO and 800 LFO, respectively. The digits in the symbol indicate the calcination temperature, while LFO means LaFeO_3_.

### 2.3. Synthesis of Monolithic Catalysts

The mixture of 7 wt% carboxymethyl cellulose (binder), 3 wt% glycerol (moisturizer), 41 wt% kaolinite (carrier), 7 wt% active carbon powder (perforation agent), 5 wt% LaFeO_3_ powder prepared at different temperatures and 30 mL of distilled water was prepared and stirred for 30 min. The mixture was kneaded for 30 min to form a dough and then wrapped with PE thin films to age under dark conditions for 12 h (this can enhance the uniformity of the distribution of soluble active components in the clay degree and enhance the catalytic activity of the molding catalyst). After aging, the dough was extruded using a mold and then placed in an electric heating blast drying oven at 100 °C for 30 min. The dried sample was then placed into a muffle furnace and warmed to 280 °C at a heating rate of 5 °C/min for 2 h. Then, the monolithic LaFeO_3_ catalyst was obtained after cooling to ambient temperature naturally. The monolithic catalyst is a cylinder with a diameter of 46 mm and a height of 15 mm, with a total of 12 through-holes, each with a diameter of 5 mm. Finally, the specific preparation path is shown in Figure 1.

### 2.4. Characterizations

Powder X-ray diffraction patterns were recorded using a Rigaku Smart Lab diffractometer with Cu Kα (λ = 1.5418 Å, 40 kV, 100 mA) at a scanning speed of 5°/min in the 2θ range of 10–80°. X-ray photoelectron spectra (XPS) were obtained with a KRATOS AXIS SUPRA. Scanning electron microscopy (SEM) were used to confirm the microstructure and morphology with a JSM-6510. 

The catalyst pore structure, pore size distribution were assessed by N_2_ adsorption and desorption test at −196 °C using a Micromeritics ASAP 2020 instrument. Total pore volume (Vp) was determined from the amount adsorbed at a relative pressure of around 0.99. The pore size distribution and average pore diameter (Dp) were determined by the BJH (Barrett–Joyner–Halenda) method from the adsorption branch of the isotherm. 

### 2.5. Catalytic Oxidation of Toluene

**Figure 2:**
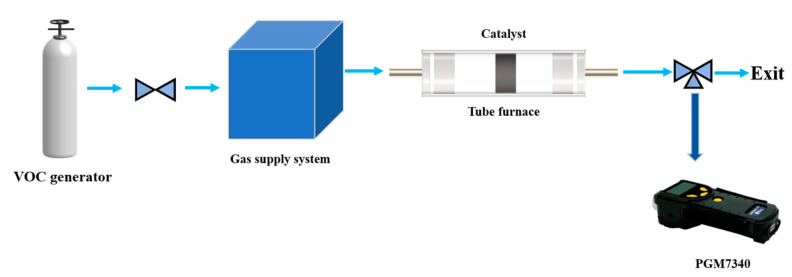
Schematic diagram of catalytic oxidation of toluene.

Figure 2 is a graphical representation of the toluene catalytic system. Air diluted toluene gas (101 ppm) from the storage tank is fed into the gas supply system using a gas flow meter to maintain an inlet toluene concentration of 101 ppm when entering the tube furnace. The diluted toluene gas and the air are mixed in the gas supply system and then enter the stationary reactor. The diluted toluene gas entering the stationary reactor reacts with oxygen on the monolithic LaFeO_3_ catalyst. The rear gas was collected using a gas sampling bag. The toluene concentration for the inlet and outlet gas was measured using a Honeywell-Warren PGM-7340ppb RAE3000 VOC gas detector. The weight hourly space velocity of inlet toluene gas is 36,000 mL/(g∙h). The conversion of toluene can be calculated as follows:

φ(C_7_H_8_) (%) = 100 × ([C_7_H_8_]_in_ − [C_7_H_8_]_out_)/[C_7_H_8_]_in_ (1)

where [C_7_H_8_]_in_ (%) and [C_7_H_8_]_out_ (%) denote the inlet and outlet toluene concentrations, respectively.

## 3. Results and Discussion

### 3.1. XRD Characterization

**Figure 3:**
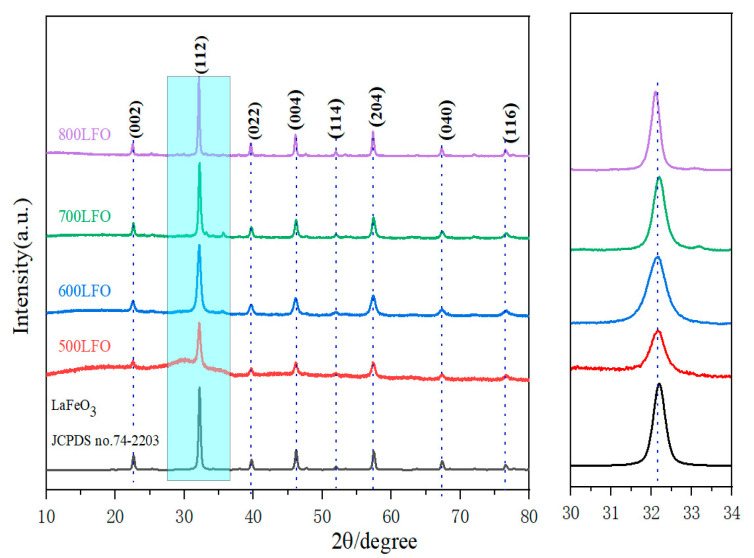
Powder X-ray diffraction patterns of LaFeO_3_ powders calcinated at different temperatures.

Figure 3 shows the powder X-ray diffraction patterns of the synthesized LaFeO_3_ powders at different calcination temperatures (500 °C, 600 °C, 700 °C and 800 °C). The XRD patterns match the standard diffraction pattern of the orthorhombic phase (JCPDS No. 74-2203). The sharp diffraction peaks indicate that LaFeO_3_ has a high crystallinity. No signals of other diffraction peaks were found, indicating that the LaFeO_3_ powders were successfully synthesized at these temperatures. The reflections located at 22.61°, 32.17°, 39.70°, 46.16°, 51.95°, 57.41°, 67.30° and 76.53° correspond to (002), (112), (022), (004), (114), (204), (040) and (116) reflection plane, respectively. Meanwhile, the X-ray diffraction patterns showed that the reflection peaks of LaFeO_3_ became sharper with the increase in calcination temperature, indicating improved crystallinity. According to Scherrer’s formula, the particle size of 500 LFO particles is 156 nm, the particle size of 600 LFO particles is 168 nm, the particle size of 700 LFO particles is 266 nm and the particle size of 800 LFO particles is 278 nm.

### 3.2. Morphology Analysis

**Figure 4:**
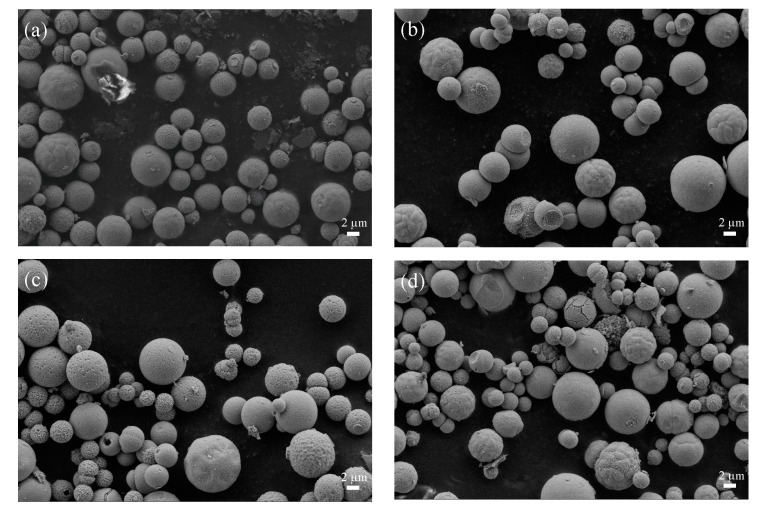
Scanning electron microscope image of 500 LFO, 600 LFO, 700 LFO and 800 LFO catalysts at 5000 magnification; (**a**) 500 LFO, (**b**) 600 LFO, (**c**) 700 LFO and (**d**) 800 LFO.

**Figure 5:**
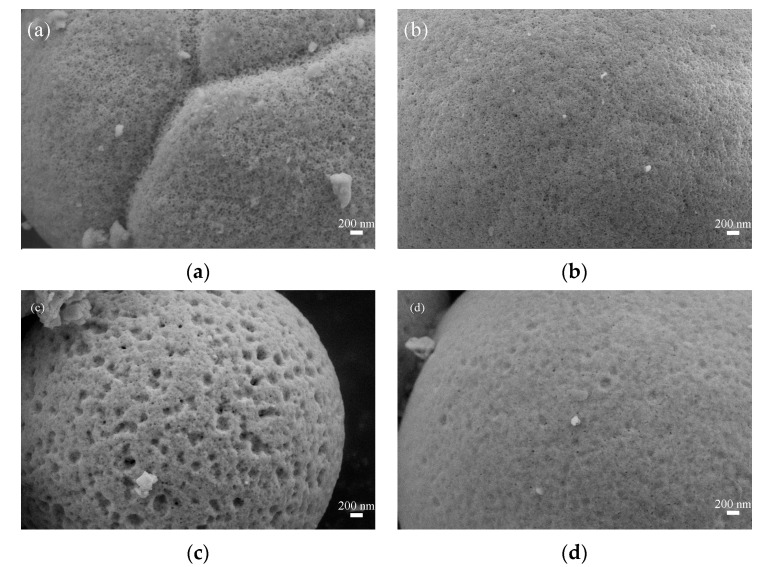
Scanning electron microscope image of 500 LFO, 600 LFO, 700 LFO and 800 LFO catalysts at 50,000 magnification; (**a**) 500 LFO, (**b**) 600 LFO, (**c**) 700 LFO and (**d**) 800 LFO.

The morphological characteristics of the LaFeO_3_ powders were analyzed by SEM. Figure 4 and Figure 5 show the 5000 and 50,000 magnification images of LaFeO_3_ synthesized at different calcination temperatures. Figure 4 shows that the morphology of LaFeO_3_ synthesized at four temperatures is characterized by spherical particles about 2–3 μm in diameter with pits and pores on the surface. The grain size derived from the SEM image is basically the same as that calculated earlier. It can be seen from Figure 5 that with the increase in calcination temperature, the surfaces of microspheres gradually become smooth and flat, the pore size becomes smaller and the number of pores increases. When the calcination temperature increases to 800 °C, the surface of microspheres gradually becomes smooth, the pores gradually decrease and it can be clearly observed that the calcination temperature changes the morphological structure of these microspheres.

### 3.3. BET Measurements

**Figure 6:**
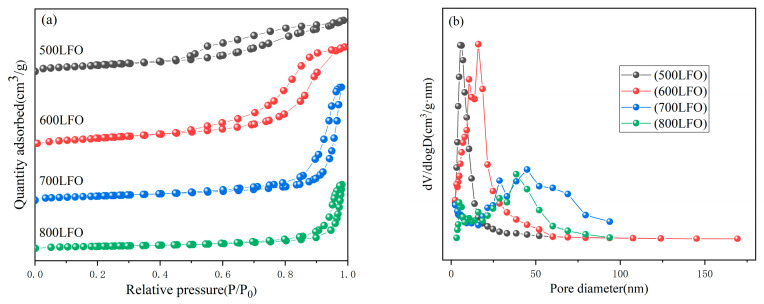
N_2_ adsorption–desorption isotherms (**a**) and pore size distribution curves (**b**) of LaFeO_3_ synthesized at different calcination temperatures.

In order to investigate the effect of calcination temperature on the specific surface area and pore size distribution of LaFeO_3_, the prepared catalysts were tested for N_2_ adsorption–desorption. The nitrogen adsorption–desorption curves and BJH pore size distribution curves are shown in Figure 6, and the specific data are shown in Table 1. The nitrogen adsorption–desorption curves of the LaFeO_3_ catalysts are type IV isothermal curves with H4−type hysteresis loops, and the pore size distribution plots better prove the existence of mesoporous structures for the four of LaFeO_3_ catalysts [37]. For the other three catalyst groups, 700 LFO has the largest specific surface area, followed by 600 LFO. The pore size distribution of the four LaFeO_3_ catalysts was found to be in a wide range based on the Barrett–Joyner–Halenda (BJH) method calculation in Figure 6b, with the pore size of the 500 LFO catalyst being around 2.7–52 nm, the 600 LFO catalyst being around 2.7–170 nm, the 700 LFO catalyst being around 2.7–52 nm and the pore size of the 800 LFO catalyst around 2.2–93 nm. It is well known that the specific surface area size of catalysts has a large influence on the catalytic activity, and a larger specific surface area exposes more active sites on the surface, prompting more pollutant molecules to be adsorbed on the catalyst surface. The results showed that 700 LFO had the largest specific surface area of 23.41 m^2^/g.

### 3.4. Structural Composition Analysis of Materials

**Figure 7:**
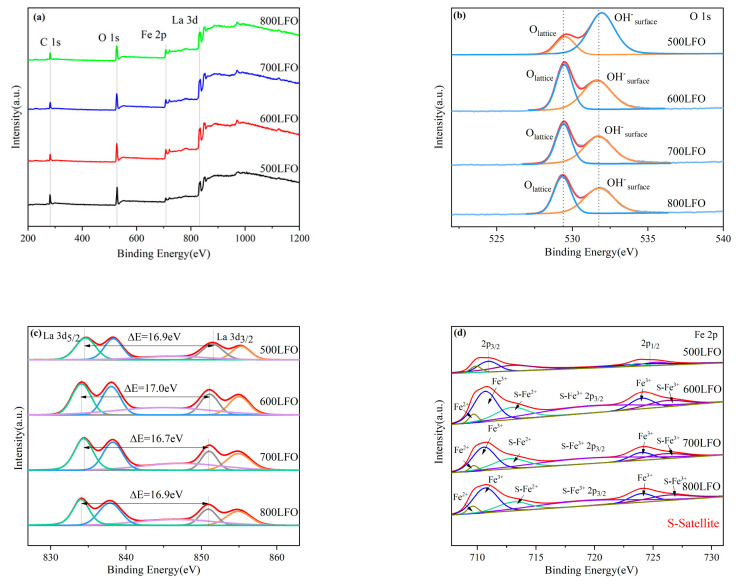
XPS diagram of 500 LFO, 600 LFO, 700 LFO and 800 LFO catalysts, and (**a**–**d**) show the complete XPS spectra, narrow spectra of O 1s, Fe 2p and La 3d energy levels of LaFeO_3_, respectively.

In order to determine the surface elemental composition and oxidation state of several samples, surface X-ray photoelectron spectroscopy was performed to analyze the LaFeO_3_. Figure 7 shows the full XPS spectra of synthetic LaFeO_3_ at different calcination temperatures (Figure 7a) and narrow spectra of O1s, Fe 2p and La 3d energy levels (Figure 7b–d). In addition, the C 1s binding energy of 284.8 eV is often used as a charge correction for other elements [38].

The high−resolution spectra of O 1s are shown in Figure 7b. For 500 LFO, the binding energy at 529.6 eV and 531.9 eV is assigned to lattice oxygen (La-O and Fe-O) and adsorbed oxygen on the surface of chemically absorbed hydroxyl or carbonate groups [39,40]. For 600 LFO, the binding energy at 529.5 eV and 531.8 eV is assigned to lattice oxygen (La-O and Fe-O) and adsorbed oxygen on the surface of chemically absorbed hydroxyl or carbonate groups. For 700 LFO, the binding energy at 529.4 eV and 531.7 eV is assigned to lattice oxygen (La-O and Fe-O) and adsorbed oxygen on the surface of chemically absorbed hydroxyl or carbonate groups. For 800 LFO, the binding energy at 529.3 eV and 531.6 eV binding energy is assigned to lattice oxygen (La-O and Fe-O) and adsorbed oxygen on the surface of chemically absorbed hydroxyl or carbonate groups. Comparing the four catalysts, the binding energy peak of O 1s shifts to lower binding energy with increasing calcination temperature, which proves that oxygen ions contribute significantly to the electron transfer within LaFeO_3_ [41]. Among the four LaFeO_3_ catalysts, 700 LFO has a higher O_ads_ (adsorbed oxygen)/O_latt_ (surface lattice oxygen) value (1.62), and the specific O_ads_/O_latt_ values are shown in Table 2.

In Figure 7c, the two sets of peaks in 500 LFO are at 834.6, 838.4 eV and 851.5, 855.2 eV, respectively, the two sets of peaks in 600 LFO are located at 834.2, 838.1 eV and 851.1, 855 eV, respectively, and the two sets of peaks in 700 LFO are at 834.3, 838.2 eV and 851, 854.9 eV, with the two sets of peaks in 500 LFO, 600 LFO and 700 LFO correlated with the spin-orbit splitting characteristic binding energy of La 3d_5/2_ and La 3d_3/2_ of La^3+^, while the peaks in La 3d_5/2_ and La 3d_3/2_ of 800 LFO are located at 834, 837.8 eV and 850.9, 854.8 eV. These two correlated peaks in each spin orbit are further split into two parts, attributed to the presence of energy loss phenomena or electron configuration mixtures caused by strong O 2p-La 4f charge events (“shock-excited” satellites) [42]. Moreover, the splitting voltages corresponding to 500 LFO, 600 LFO, 700 LFO and 800 LFO are 16.9 eV, 17 eV, 16.7 eV and 16.9 eV.

In the Fe 2p spectrum of Figure 7d, the peaks at 710.9 and 725.2 eV and two satellite peaks (714.9 and 722.4 eV) in the 500 LFO indicate the presence of Fe^3+^ in the LaFeO_3_, and the binding energies at 709.9 and 713.1 eV are consistent with the Fe^2+^ match in the LaFeO_3_. The two satellite peaks (716.1 and 723.1 eV) of the peak at 710.7 and 724.2 eV in 600 LFO indicate the presence of Fe^3+^ in LaFeO_3_, and the binding energy at 709.7 and 713.1 eV indicates the presence of Fe^2+^ in LaFeO_3_. The two satellite peaks of the peak at 710.6 and 724 eV (716.2 and 723 eV) in the 700 LFO indicate the presence of Fe^3+^ in the LaFeO_3_, and the characteristic binding energy at 709.7 and 712.9 eV indicates the presence of Fe^2+^ in the LaFeO_3_. The two satellite peaks of the peak at 710.7 and 724.2 eV in the 800 LFO (715.9 and 723.2 eV) indicate the presence of Fe^3+^ in the LaFeO_3_, and the characteristic binding energies at 709.6 and 713.2 eV indicate the presence of Fe^2+^ in the LaFeO_3_. This implies that two mixed Fe^2+^/Fe^3+^ states exist in the octahedra of LaFeO3 sharing prismatic positions, while the presence of Fe^2+^ oxidation states can be attributed to the generation of oxygen vacancies. In addition, the spin-orbit splitting of the 500 LFO, 600 LFO, 700 LFO and 800 LFO samples was 13.6 eV, 13.5 eV, 13.4 eV and 13.5 eV, respectively. Finally, the 700 LFO catalyst had the highest surface Fe^2+^/Fe^3+^ molar ratio (0.78), and the order of Fe^2+^/Fe^3+^ molar ratios for the four catalyst groups was: 700 LFO > 800 LFO > 600 LFO > 500 LFO. The specific Fe^2+^/Fe^3+^ molar ratios are shown in Table 2. From XPS, the content ratios of Fe, La and O in 500 LFO were 6.2%, 12.9% and 80.9%, respectively. The content ratios of Fe, La and O in 600 LFO were 11.1%, 15.4% and 73.5%, respectively. The content ratios of Fe, La and O in 700 LFO were 9.6%, 16.7% and 73.7%. The content ratios of Fe, La and O in 800 LFO were 11.2%, 14.7% and 74.1%, respectively.

### 3.5. Catalytic Performance

**Figure 8:**
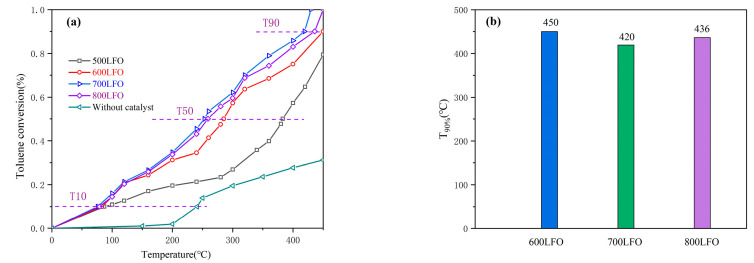
(**a**) Toluene conversions as function of reaction temperature over LaFeO_3_ perovskite catalysts sintered at different temperatures (**b**) The temperature required for each catalyst at a conversion of T_90%_ for toluene.

The catalytic performance of the prepared sets of monolithic catalysts for toluene was investigated below 450 °C. Figure 8a shows the performance of the four sets of monolithic LaFeO_3_ catalysts for toluene oxidation at a toluene concentration of 101 ppm and an air rate of 100 mL/min (WHSV = 36,000 mL/(g∙h)). Figure 8b shows the reaction temperatures corresponding to the conversion of toluene at 90% for the three LaFeO_3_ catalysts. It is well known that catalysts for catalytic oxidation performance of gases are usually evaluated using T_10%_, T_50%_ and T_90%_ (corresponding to the reaction temperatures required to reach 10%, 50% and 90% toluene conversions). 

According to Figure 8a, the monolithic catalysts with the addition of LaFeO_3_ all showed higher catalytic activity than the monolithic catalysts without the addition of LaFeO_3_. The catalytic activities of the monolithic catalysts with LaFeO_3_ were ranked as follows: 700 LFO > 800 LFO > 600 LFO > 500 LFO. Obviously, the monolithic catalysts without LaFeO_3_ did not show high degradation of toluene, and the thermal degradation of toluene was only 31.2% at 450 °C, while 700 LFO with LaFeO_3_ catalyst showed the best catalytic activity for toluene with T_10%_, T_50%_ and T_90%_ of 76 °C, 253 °C and 420 °C, respectively, which were 7 °C, 32 °C and 30 °C lower than those of 600 LFO with T_10%_, T_50%_ and T_90%_, respectively. Table 3 shows the reaction temperatures of the monolithic catalyst with and without the addition of LaFeO_3_ at 10%, 50% and 90% conversion of toluene. Undoubtedly, the specific surface area, pore structure, surface active center and surface adsorbed oxygen concentration of the catalysts are significantly related to their catalytic activity. From the experimental data and characterization, it is clear that 700 LFO has the largest specific surface area, the most active centers and the most abundant surface adsorbed oxygen concentration compared to 500 LFO, 600 LFO and 800 LFO, which results in better catalytic performance in the toluene oxidation experiments.

**Figure 9:**
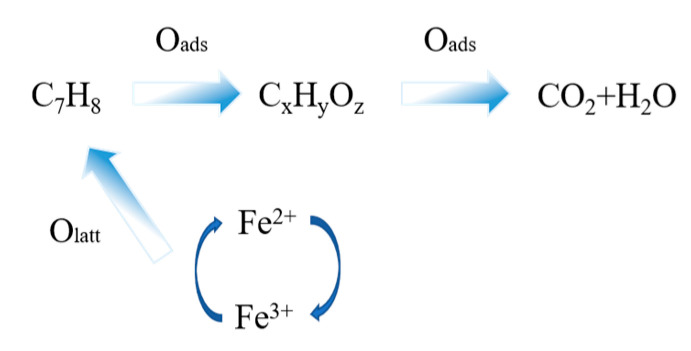
Catalytic oxidation of toluene mechanism diagram.

Figure 9 demonstrates the reaction mechanism of catalytic oxidation of toluene gas by the LaFeO_3_ catalyst. Fe^2+^ is trapped by oxygen molecules when transferring electrons, and oxygen molecules are adsorbed to oxygen vacancies and activated. Negatively charged oxygen anions (O^−^, O^2−^) fill the oxygen vacancies and provide oxygen resources for the catalytic reaction. At the same time, the transfer of electrons leads to the transformation of Fe^2+^ to Fe^3+^. Toluene molecules are adsorbed on Fe^3+^, and the coordination between them allows electrons to be transferred from the toluene to the Fe^3+^ position. Regeneration of Fe^2+^ and positively charged toluene occurs due to electron transfer. Negatively charged oxygen and positively charged toluene react to produce carbon dioxide and water through the breaking of old chemical bonds and the formation of new ones [43].

## 4. Conclusions

Porous powder LaFeO_3_ was readily prepared using the conventional high temperature calcination of precursors obtained from hydrothermal syntheses. Finally, the honeycomb LaFeO_3_ catalysts were prepared by the one-shot molding method. From the experimental results, it was shown that the calcination temperature from 500 °C to 800 °C had a significant effect on the morphology, structure, specific surface area and the concentration of adsorbed oxygen on the surface of the LaFeO_3_ catalysts. According to the SEM results, comparing the morphology of the other three groups of LaFeO_3_ catalysts, the 700 LFO catalyst has more pits on the surface, forming more pores and smaller pores, resulting in more active sites exposed on the catalyst surface, which has a greater impact on the performance of the porous catalyst. Among the prepared groups of LaFeO_3_ catalysts, 700 LFO showed the largest specific surface area (23.41 m^2^/g), the more abundant concentration of surface adsorbed oxygen as well as the best catalytic performance for toluene (T_10%_, T_50%_ and T_90%_ at a toluene concentration of 101 ppm and an air rate of 100 mL/min (WHSV = 36,000 mL/(g∙h)), respectively 76 °C, 253 °C and 420 °C).

## Figures and Tables

**Table 1:** BET analysis of LaFeO_3_ synthesized by different calcination temperatures.

Catalysts	BET Surface Area (m^2^/g)	Average Pore Size (nm)	Total Pore Volume (cm^3^/g)
500 LFO	13.42	8.99	0.04
600 LFO	18.91	13.66	0.08
700 LFO	23.41	27.32	0.09
800 LFO	6.03	33.12	0.05

**Table 2:** XPS parameters of different elements in LaFeO_3_ catalysts synthesized at different calcination temperatures.

Catalysts		Binding Energy (eV)							
	La^3+^	La^3+^	Fe^3+^	Fe^3+^	Fe^2+^	O_ads_	O_latt_	Fe^2+^/Fe^3+^	O_ads_/O_latt_
	3d 5/2	3d 3/2	2p 3/2	2p 1/2	2p 3/2	1 s	1 s		
500 LFO	834.2/838.4	851.5/855.2	710.9/719.8	724.5/725.5	709.9/713.1	529.6	531.9	0.68	2.63
600 LFO	834.1/838.1	851.1/855	710.7/719.5	724.2/726.3	709.7/713.1	529.5	531.8	0.70	1.21
700 LFO	834.3/838.2	851/854.9	710.6/719.3	724/726.5	709.7/712.9	529.4	531.7	0.78	1.62
800 LFO	834/837.8	850.9/854.8	710.7/719.1	724.2/726.7	709.6/713.2	529.3	531.6	0.71	1.26

O_ads_ stands for adsorbed oxygen. O_latt_ stands for surface lattice oxygen.

**Table 3:** Reaction temperature of the different LaFeO_3_ catalyst for toluene conversion up to T90%.

Catalysts	Toluene Oxidation Activity (°C)		
	T_10%_	T_50%_	T_90%_
500 LFO	87	383	/
600 LFO	83	285	450
700 LFO	76	253	420
800 LFO	79	259	436
Without catalyst	241	/	/

## Data Availability

The data presented in this study are available on request from the corresponding author.
